# Efficacy of Bilateral Transversus Abdominis Plane and Ilioinguinal-Iliohypogastric Nerve Blocks for Postcaesarean Delivery Pain Relief under Spinal Anesthesia

**DOI:** 10.1155/2018/1948261

**Published:** 2018-01-21

**Authors:** Seid Adem Ahemed, Zewditu Abdissa Denu, Habtamu Getinet Kassahun, Demeke Yilikal Fentie

**Affiliations:** Department of Anesthesia, College of Medicine and Health Sciences, University of Gondar, Gondar, Ethiopia

## Abstract

**Background:**

Caesarean delivery can be associated with considerable postoperative pain. While the benefits of transversus abdominis plane (TAP) and ilioinguinal-iliohypogastric (II-IH) nerve blocks on pain after caesarean delivery via Pfannenstiel incision have been demonstrated, no enough investigations on the comparison of these blocks on pain after caesarean delivery have been conducted in our setup.

**Method:**

An institutional-based prospective observational cohort study was conducted to compare the analgesic efficacy of those blocks. We observed 102 postoperative parturients. The outcome measure was the severity of pain measured using a numeric rating scale.

**Result:**

Twenty-four hours after surgery, the NRS score at rest was (0.90 ± 0.80) versus (0.67 ± 0.58) and at movement (1.2 ± 1.07) versus (0.88 ± 0.76) for the TAP and II-IH groups, respectively. Twenty-four hours after surgery, the mean tramadol consumption was (55.45 ± 30.51) versus (37.27 ± 27.09) mg in TAP and II-IH groups, respectively (*p* = 0.009). The mean first analgesic requirement time was also prolonged in the II-IH group.

**Conclusion and Recommendations:**

There was no statically significant difference between TAP and II-IH blocks regarding postoperative pain score, but the II-IH block significantly reduced the total tramadol consumption and prolonged the time to first analgesic request than TAP. Thus, we recommend the II-IH nerve block.

## 1. Introduction

Pain management is crucially important in the postoperative period as it increases patient comfort and satisfaction [1]. Caesarean delivery (CD) has been one of the most frequently performed major surgical interventions and causes severe postoperative pain [2]. Caesarean delivery and subsequent manipulation performed through Pfannenstiel incision are associated commonly with a significant degree of pain in the postoperative period; 79% of women experience pain at the incision site that can last for up to 2 months [3].

Inadequate postoperative analgesia is one of the most common causes for poor patient satisfaction following caesarean delivery [3, 4].

Childbirth is an emotion-filled event, and the mother wants to bond with her newborn as early as possible. Inadequate postoperative pain relief after CD can negatively impact ambulation, breastfeeding, and even maternal bonding [2, 5].

Poor pain control in the postoperative period can lead to chronic pain syndromes and poor quality of life [2, 5].

The provision of effective postoperative analgesia is a key to facilitate early mobilization of the mother, infant care, and prevention of postoperative morbidity. Improvement in postoperative analgesia may not only increase patient satisfaction but also diminish the duration of hospital stay and reduce the risk of complications [6, 7].

The ideal form of postoperative analgesia is unknown, but many procedures are carried out under spinal anesthesia, and currently, opioids are commonly used for relief of postoperative pain after caesarean section, either by intrathecal administration prior to section or by postoperative parenteral administration as a component of multimodal analgesia during the postoperative period [6, 8].

Even if opioids are available to be administered via the spinal or systemic route, they had adverse effects such as nausea, vomiting, sedation, itching, and risk of delayed maternal respiratory depression, all of which reduce overall patient satisfaction [2, 3, 8].

Additionally, these opioid-related adverse effects can produce other problems for new mothers such as delayed initiation of breastfeeding and impairment of mother/infant bonding [3].

An ideal method of pain relief after caesarean delivery should be cost-effective, safe for the mother, require minimal monitoring, and use drugs that are not secreted into breast milk. Moreover, the mother should not be sedated by the drugs that prevent her from moving freely and caring for the newborn.

Minor side effects, such as pruritus and shivering, may interfere with care of the newborn, leading to less maternal satisfaction. Drug availability, maternal health conditions, patient preferences, and availability of medical expertise and trained support staff also play a role in choice of the analgesic method.

Many scholars have been studying to find the safest and effective way of interventions for postoperative CD pain management, and they suggest methods like opioid or local anesthetic skin infiltration, epidural analgesia, intrathecal or intravenous opioids, and abdominal field blocks like TAP and II-IH.

Among the above-listed ways of pain management, intravenous opioids and regional nerve blocks are the mainstay of treatment for postcaesarean pain here in the study area.

Epidural analgesia, which is the gold standard for control of labour pain and postoperative pain, is not commonly practiced in our setup due to lack of the epidural kit.

Abdominal field blocks like TAP and II-IH are the mainstay of treatment for postcaesarean pain for both the midline and Pfannenstiel incision because of the opioid sparing effect, prolonged pain relief, and technical simplicity, and also, it does not need repeated injection for optimal pain relief [3, 9].

TAP and II-IH nerve blocks are well known and easy to perform, and currently, these blocks are done in the study area for post-CD pain management.

There are no enough data concerning the efficacy of TAP versus II-IH nerve blocks in the management of postoperative pain in parturients undergoing caesarean section.

Most of the studies that have been done to determine the efficacy of those blocks in post-CD pain management are done in western countries, but there is no published literature in Ethiopia.

The presence of racial, cultural, genetic, and sociodemographic differences in the perception of pain has been well documented [10, 11].

There is also controversy regarding the efficacy of the two blocks [12, 13], so comparing the effectiveness of TAP blocks with that of II-IH nerve blocks will help us to have a best practice to the study area, and knowing the efficacy of these blocks will allow anesthetists to choose the most effective one to manage postoperative CD pain.

The aim of this study was to compare the efficacy of the TAP block and II-IH block in controlling postcaesarean section pain.

## 2. Methods

An institutional-based observational cohort study was conducted from April 1 to May 30, 2017, at Gondar University Teaching Hospital after we secured ethical approval from the University of Gondar Ethical Review Board. The minimum sample size calculated for this study was 102, and the sample size calculation was done based on the two population proportion principles:

(1) $$\begin{array}{c} \frac{p_{1}\left(1-p_{1}\right)+p_{2}\left(1-p_{2}\right)}{{\left(p_{2}-p_{1}\right)}^{2}}\times f\left(\alpha ,\beta \right). \end{array}$$

- Hence, the incidence of moderate-to-severe pain after CD without intervention was 87%, and we got pain reduction after clients received bilateral TAP and II-IH blocks 40% and 70%, respectively. And we calculated *p*_1_ by reducing 40% of 87% from 87% (*p*_1_ = 0.87−0.348 = 0.522) and *p*_2_ by reducing 70% of 87% from 87% (0.87−0.69 = 0.261) since we need only pain reduction after the block from the incidence of pain (87%) by taking $\dot{f}$(*α*, *β*) = 7.85 with a power of 80% and 0.05 significance.
- Since *f*(*α*, *β*) = 7.85 or 10.5 for 80% or 90% power, respectively, with 5% significance, the significance (risk of type I error) is almost always set at 5%.

So per group, we have 51 participants.

We included ASA I and ASA II patients. Finally, we selected every consecutive parturient for whom the abovementioned nerve blocks were done and who also volunteered to give consent to participate in the study till the required sample is achieved. Our primary outcome measure was the severity of pain which was measured using the numeric rating scale. The secondary outcomes were the total analgesic consumption and time for the first analgesic request. As to the data collection procedure, two trained anesthetists collect all the required information based on the checklist prepared in English. The investigators had no power to decide which type of block would be given for the mother (only the responsible anesthesiologist had this right, i.e., he/she did the block as he/she wishes, either TAP or II-IH).

But the investigator stayed in the operating theater and saw the type of block that the responsible anesthesiologist had done and put the code on the chart. The type of block was not clearly recorded on the chart except the code so that data collectors are blinded to the type of block done for each mother.

The relative proportion of these blocks in our institution looks equal (1 : 1) because everybody is doing these blocks randomly as they wish for transverse incision, but for vertical incision, they do only TAP.

The block was done by using the landmark technique for both types, and a total of 32 ml of 0.25% bupivacaine for II-IH block (8 ml in each side) and 40 ml of 0.25% bupivacaine for TAP block (20 ml in each side) were given.

Pain assessment was performed at 0 hr, 4 hr, 6 hr, 8 hr, 12 hr, and 24 hr in the ward by blinded data collectors who were unaware of the type of the nerve block done. And the total analgesic consumption within 24 hours was also recorded.

Data were coded, entered, cleaned, and cross-checked with SPSS version 20 statistical package. The data were tested for normality using the Shapiro–Wilk normality test. Normally distributed data were analyzed using Student's *t*-test.

All data other than categorical parameters were analyzed using Student's *t*-test.

The comparisons of categorical parameters were analyzed using the chi-square test or Fisher's exact test as required and expressed in % and numbers. Data were presented as mean ± SD. *p* value < 0.05 was considered statistically significant.

## 3. Results

A total of hundred and two clients were enrolled in the current study with a response rate of 100%. The age of participants was 26.98 with a standard deviation of 2.4 years. Assessment of the ASA physical status showed that 78% of the TAP and 76% of the II-IH groups were ASA I and 21.56% of the TAP and 23.5% of the II-IH groups were ASA II (Table 1).

Postoperative vital signs (postoperative pulse rate and mean arterial blood pressure) were comparable between the two groups (Tables 2 and 3).

### 3.1. Postoperative Pain Scores using Numerical Rating Scale

With regard to the postoperative pain score, there was no difference between the two groups. We assessed the pain score at rest, on coughing, and at movement. The result shows no statistically significant difference between the two groups (Tables 4 and 5).

The mean tramadol consumption for the TAP group was 52.45 with a standard deviation of 30.5, and for the II IH group, it was 37.25 ± 27.09 mg; the mean first analgesic consumption time in this study was 10.71 ± 7.67 hr for the TAP group and 14.09 ± 8.20 hr for the II-IH group, with a *p* value of 0.03 (Table 6)

## 4. Discussion

We found that there was no statistically significant difference between TAP and II-IH blocks in the numeric pain rating score both at rest and at movement for the first postoperative 24 hours, but the II-IH nerve block significantly reduced total tramadol consumption and prolonged the time for the first analgesic request.

Studies comparing the two blocks are rare, so we compare our results separately for each block.

Our result was comparable with the randomized controlled study done in Russia which showed that there was no statistically significant VAS (visual analogue score) difference between TAP and II-IH blocks after caesarean delivery via Pfannenstiel incision within 24 hours postoperatively [14].

A randomized comparative study done in New Zealand showed that the II-IH nerve block reduced the pain score significantly and postoperative total tramadol consumption (*p*=0.03) than the TAP block after inguinal surgery [12]. This is consistent with our result regarding tramadol consumption.

A systematic review and meta-analysis done by Abdallah et al. in Canada demonstrated that the TAP block enhances analgesia after caesarean delivery with detectable analgesic effects for the entire 24 hours [5].

Another review and meta-analysis done by Mishriky et al. concluded that the TAP block improved postoperative analgesia and reduced the pain score in women undergoing CD [4]. These two are in agreement with our results.

A meta-analysis done by Champaneria et al. in 2016 showed that the TAP block provided effective analgesia and reduced the postoperative pain score after caesarean section [15]. This is comparable to our result.

Our result is comparable with a randomized control trial study done in Saudi Arabia that showed the NRS score was significantly lower in the TAP group than the control up to 24 hr both at rest and at movement after caesarean delivery via Pfannenstiel incision [15].

On the other hand, a study done by Sakalli et al. showed that the II-IH nerve block decreased the mean VAS score both at rest and at movement within 24 hours after CD [2]. This is comparable with our finding.

Similarly, a study done in Jordan showed a significant reduction in the mean VAS score after the II-IH nerve block when compared with the placebo group in parturients who underwent caesarean delivery under general anesthesia [16, 17].

Our study demonstrated that II-IH reduced the NRS pain score which is consistent with a study done by Bunting and McConachie and Ganta and colleagues, who analyzed the mean VAS score, and they found it to be less with the II-IH block as compared with the placebo group in parturients who underwent caesarean delivery [18]. A study of Bell and colleagues also demonstrated that the VAS score was reduced with the II IH nerve block [3].

A study done in Turkey by Yucel and colleagues showed that the II-IH nerve block reduced the VAS score for the first 24 hr postoperatively than the control group. This is comparable with the current study [19].

In this study, the mean time for the first analgesic request was significantly prolonged in the II-IH group (*p*=0.03). This is consistent with the finding of the previous comparative study done in Russia which showed that the II-IH block prolonged the time to first analgesic requirement in a statistically significant fashion than the TAP block following CD [14].

We found that the total amount of tramadol consumption over the first 24-hour postoperative time was lower in the II-IH group than that in the TAP group. This result is comparable with the previous study [14]. One study done in New Zealand was also in line with this study [12].

Our finding was also comparable with the studies conducted by Yucel et al. and Naghshineh et al. where they have found that postoperative analgesic consumption was significantly lower in the parturients who received the II-IH block as compared with the control group [20].

## 5. Conclusion and Recommendation

There was no statistically significant difference between TAP and II-IH nerve blocks regarding the postoperative pain score in each time point both at rest and at movement, but the II-IH block significantly reduced total tramadol consumption and prolonged the time to first analgesic request than TAP.

We recommend the II-IH nerve block for postcaesarean delivery pain management via Pfannenstiel incision.

## Figures and Tables

**Table 1:** Sociodemographic and other characteristics of study participants from April 1 to May 30, 2017, in Northwest Ethiopia.

Character	TAP (*n* = 51)	II-IH (*n* = 51)	*p* value
Age (years)	27.27 ± 2.88	26.69 ± 1.79	0.219
Height (meter)	1.67 ± 0.04	1.65 ± 0.04	0.077
Weight (kg)	63.10 ± 7.69	64.88 ± 9.45	0.299
BMI (kg/m^2^)	22.63 ± 2.01	23.29 ± 2.97	0.192
ASA I	40 (78.51%)	39 (76.34%)	0.814
ASA II	11 (21.56%)	12 (23.52 %)	
Parity			
Nulliparous	28 (54.90%)	27 (52.90%)	0.843
Multiparous	23 (45.09%)	24 (47.05%)	
Number of previous c/s			
0	39 (76.47%)	38 (74.50%)	
1	11 (21.56%)	9 (17.64%)	0.529
2	1 (1.96%)	3 (5.88%)	
3	0 (0%)	1 (1.96%)	
Level of sensory block			
T6-T4	7 (13.72%)	8 (15.68%)	0.780
T7-T10	44 (86.27%)	43 (84.31%)	
Duration of surgery (min)	45.29 ± 9.24	48.33 ± 7.85	0.077

**Table 2:** Postoperative pulse rate (beats per minute) in both groups who underwent caesarean delivery under spinal anesthesia in Gondar University Hospital from April 1 to May 30, 2017, in Northwest Ethiopia.

Postoperative time	TAP group (*n* = 51)	II-IH group (*n* = 51)	*p* value
0 hour	77.29 ± 4.11	75.20 ± 7.91	0.96
4 hours	76.00 ± 5.21	77.27 ± 5.37	0.22
6 hours	76.02 ± 5.98	77.35 ± 2.99	0.15
8 hours	76.04 ± 6.09	76.16 ± 6.76	0.92
12 hours	76.41 ± 3.00	76.53 ± 2.36	0.82
24 hours	76.53 ± 3.85	75.16 ± 3.63	0.06

Data are mean ± SD.

**Table 3:** Postoperative mean arterial blood pressure (mmHg) in both groups who underwent caesarean delivery under spinal anesthesia in Gondar University Hospital from April 1 to May 30, 2017, in Northwest Ethiopia.

Postoperative time	TAP group (*n* = 51)	II-IH group (*n* = 51)	*p* value
0 hour	78.43 ± 5.45	76.42 ± 5.59	0.06
4 hours	84.33 ± 8.29	84.25 ± 7.62	0.96
6 hours	85.05 ± 6.18	85.01 ± 6.47	0.97
8 hours	87.23 ± 5.43	88.08 ± 3.88	0.36
12 hours	84.77 ± 6.66	86.22 ± 6.02	0.25
24 hours	89.03 ± 3.24	89.41 ± 3.84	0.58

Data are mean ± SD.

**Table 4:** Postoperative NRS scores at rest over the first 24 postoperative hours among parturients who underwent caesarean delivery under spinal anesthesia in Gondar University Hospital from April 1 to May 30, 2017, in Northwest Ethiopia.

Character	TAP group	II-IH group	*p* value
NRS score at 0 hr	0.00	0.00	
NRS score at 4 hr	0.69 ± 1.46	0.41 ± 1.00	0.27
NRS score at 6 hr	0.67 ± 0.136	0.41 ± 1.09	0.30
NRS score at 8 hr	0.65 ± 1.18	0.35 ± 0.79	0.14
NRS score at 12 hr	0.49 ± 0.857	0.16 ± 0.46	0.22
NRS score at 24 hr	0.90 ± 0.80	0.67 ± 0.58	0.95

Data are mean ± SD.

**Table 5:** Postoperative NRS scores at movement or on coughing in the postoperative 24 hours of parturients who underwent caesarean delivery under spinal anesthesia in Gondar University Hospital from April 1 to May 30, 2017, in Northwest Ethiopia.

Character	TAP group	II-IH group	*p* value
NRS score at 0 hr	0.00	0.00	
NRS score at 4 hr	1.63 ± 2.12	0.96 ± 1.52	0.07
NRS score at 6 hr	1.84 ± 1.88	1.24 ± 1.53	0.07
NRS score at 8 hr	1.39 ± 1.53	1.00 ± 1.09	0.14
NRS score at 12 hr	1.39 ± 1.49	0.92 ± 1.197	0.08
NRS score at 24 hr	1.20 ± 1.07	0.88 ± 0.76	0.09

Data are mean ± SD.

**Table 6:** Postoperative total opioid consumption and first analgesic request time over the first 24 postoperative hours of parturients who underwent caesarean delivery under spinal anesthesia in Gondar University Hospital from April 1 to May 30, 2017, in Northwest Ethiopia.

Character	TAP group	II-IH group	*p* value
Total tramadol consumption (mg)	52.45 ± 30.51	37.25 ± 27.09	0.009
First analgesic request time (hr)	10.71 ± 7.67	14.09 ± 8.20 hr	0.03

Data are mean ± SD.
